# Supplementary material for: Microsolvation of a Proton by Ar Atoms: Structures and Energetics of ArnH+ Clusters
Source: Molecules. 2024 Aug 28;29(17):4084. doi: 10.3390/molecules29174084 (PMC11487409; doi:10.3390/molecules29174084)
Supplement: Supplementary file 1 [file molecules-29-04084-s001.zip › molecules-3153612-supplementary.pdf]

Microsolvation of a Proton by Ar Atoms: Structures and Energetics of Ar_nH^+ Clusters

María Judit Montes de Oca-Estévez [1,2,†] and Rita Prosmiti [1,*]

1 Institute of Fundamental Physics (IFF-CSIC), CSIC, Serrano 123, 28006 Madrid, Spain; juditmontesdeoca@iff.csic.es
2 Atelgraphics S.L., Mota de Cuervo 42, 28043 Madrid, Spain
* Correspondence: rita@iff.csic.es; Tel.: +34-91-5616800 (ext. 442292)
† Doctoral Programme in Theoretical Chemistry and Computational Modelling, Doctoral School, Universidad Autónoma de Madrid, 28049 Madrid, Spain.

SUPPLEMENTARY MATERIAL

Table S1: Binding energies (in cm^{-1}) from CCSD(T)/AV6Z and MP2/AVQZ calculations for $[Ar_nH]^+$ complexes, with n=1-3 and 1-9, respectively, $(V_{2B}/V_{3B})V_{4B}$ and previous theoretical work [8]. The corresponding optimized structures (side and top views) obtained from the CCSD(T)/AV6Z calculations for n=1-3 and V_{4B} potential for n=3-24 are also depicted.

	$[Ar_nH]^+$		This work		Ref. [8]
n	Side view	Top view	MP2/AVQZ<br>CCSD(T)/AV6Z	$(V_{2B}/V_{3B})V_{4B}$	-MP2/AVTZ
1	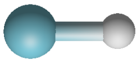	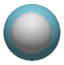	-32441.82<br>-32799.69	-	-31180
2	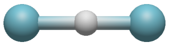	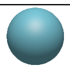	-5794.70<br>-5353.75	(-3097/-)	-5739
3	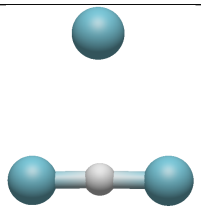	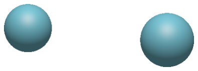	-767.20<br>-712.73	(-5295/-861)	-718
4	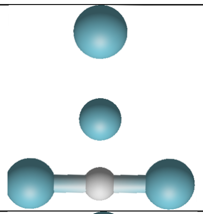	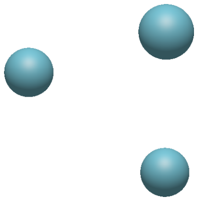	-848.09	(-5231/-867)-831.17	-796
5	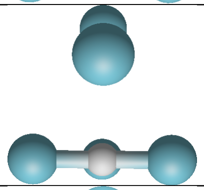	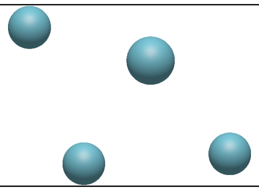	-833.81	-838.77	-784
6	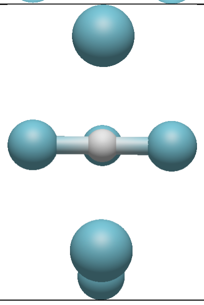	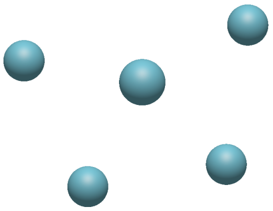	-833.41	-846.66	-777

7	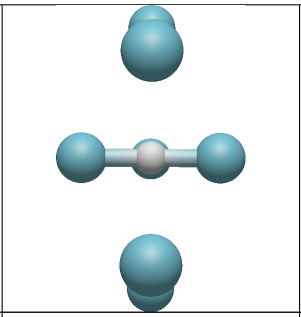	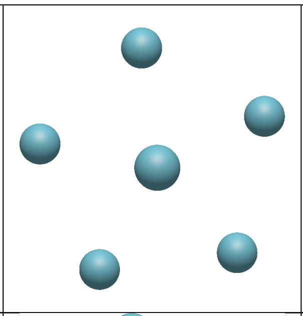	-911.34	-944.05	-855
8	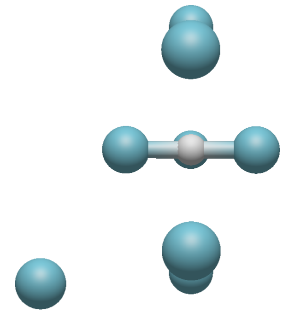	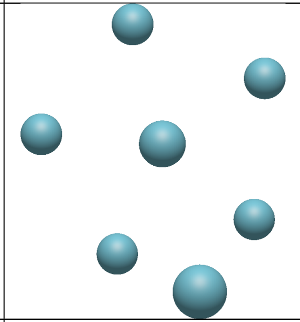	-605.46	-632.33	-557
9	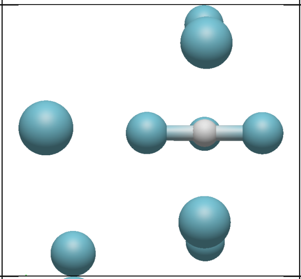	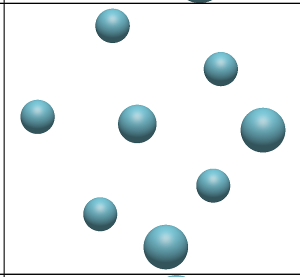	-630.05	-727.03	-
10	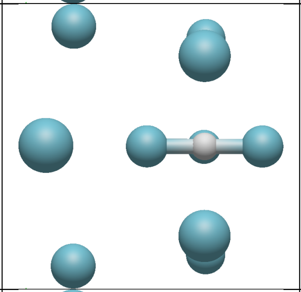	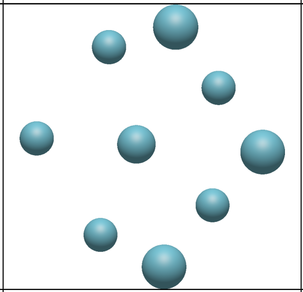	–	-736.31	-
11	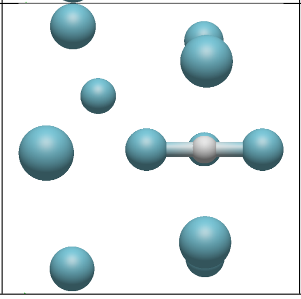	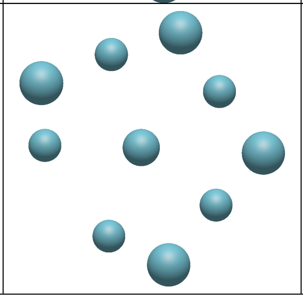	–	-746.36	-

12	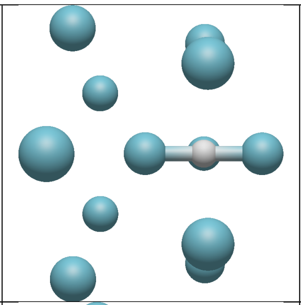	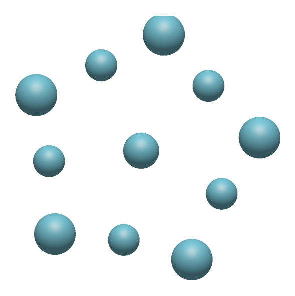	–	-825.62	-
13	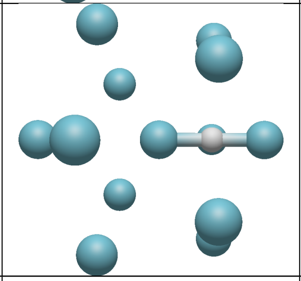	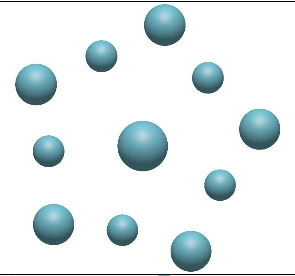	–	-992.76	-
14	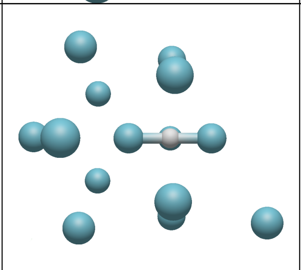	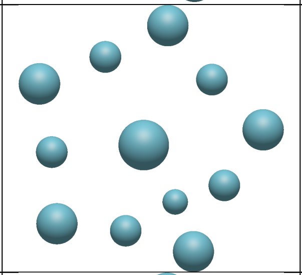	–	-638.93	-
15	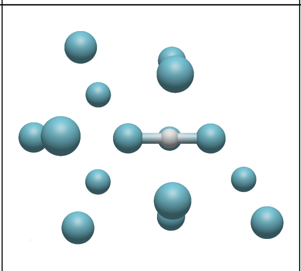	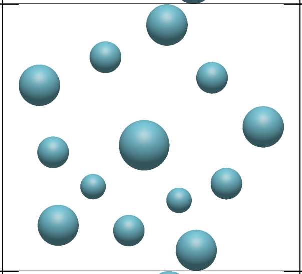	–	-741.55	-
16	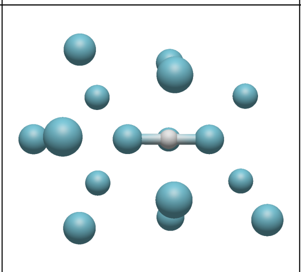	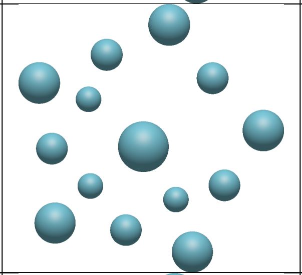	–	-747.65	-
17	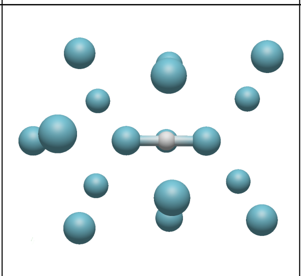	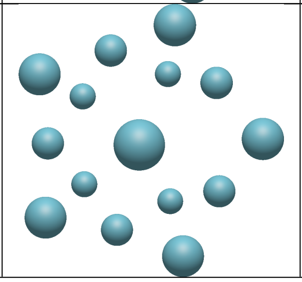	–	-757.01	-

18	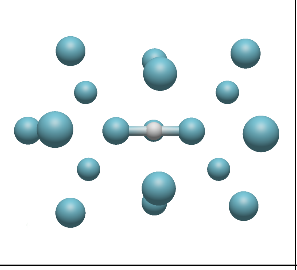	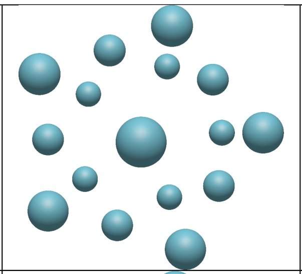	–	-694.84	-
19	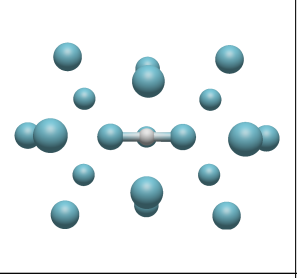	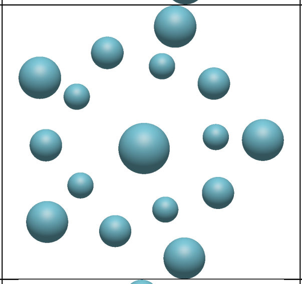	–	-1107.55	-
20	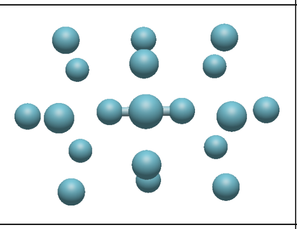	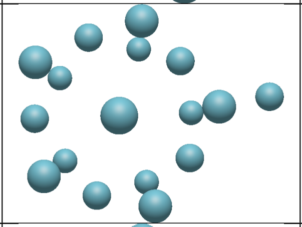	–	-429.39	-
21	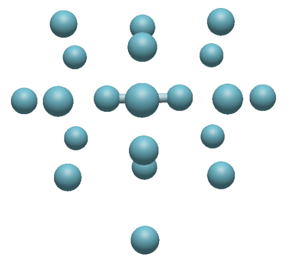	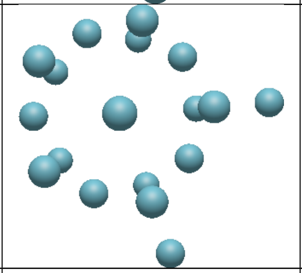	–	-632.99	-
22	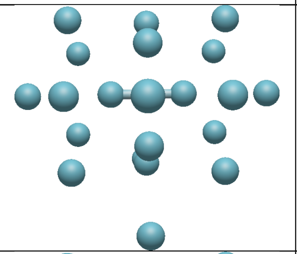	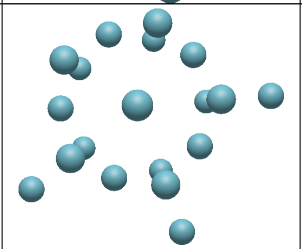	–	-472.66	-
23	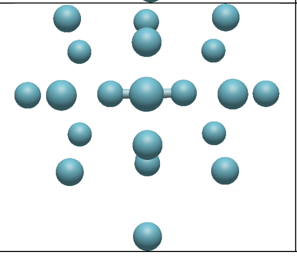	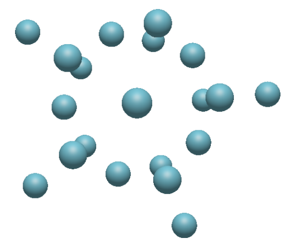	–	-563.48	-

24	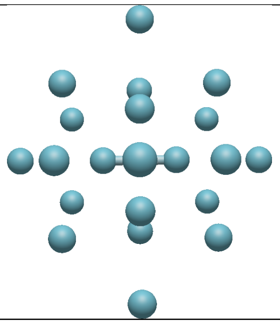	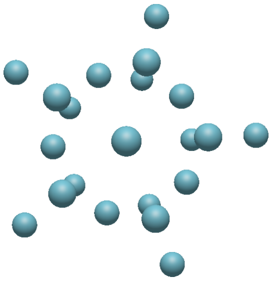	–	-513.60	-
